# Estimation of errors in diffraction data measured by CCD area detectors

**DOI:** 10.1107/S0021889810033418

**Published:** 2010-10-01

**Authors:** David Waterman, Gwyndaf Evans

**Affiliations:** a Diamond Light Source, Harwell Science and Innovation Campus, Didcot, Oxfordshire OX11 0DE, England

**Keywords:** error estimation, CCD area detectors, detector simulation, pixel correlation

## Abstract

Current methods for diffraction-spot integration from CCD area detectors typically underestimate the errors in the measured intensities. In an attempt to understand fully and identify correctly the sources of all contributions to these errors, a simulation of a CCD-based area-detector module has been produced to address the problem of correct handling of data from such detectors. Using this simulation, it has been shown how, and by how much, measurement errors are underestimated. A model of the detector statistics is presented and an adapted summation integration routine that takes this into account is shown to result in more realistic error estimates. In addition, the effect of correlations between pixels on two-dimensional profile fitting is demonstrated and the problems surrounding improvements to profile-fitting algorithms are discussed. In practice, this requires knowledge of the expected correlation between pixels in the image.

## Abstract 2

A simulation of a CCD area detector is presented. The methods for error estimation on data obtained from images from such detectors by two-dimensional integration are considered and improvements incorporating a realistic instrumental response are suggested.

## 1. Introduction   

The typical setup for macromolecular X-ray crystallography uses a large two-dimensional area detector to record accurately the positions and intensities of the diffraction spots. Many different types of two-dimensional area detector have been produced, including film, image plates, and various configurations of detectors based on CCD and CMOS sensors. Developments in X-ray detection technologies have been spurred on by the proliferation of beamlines at large-scale third-generation synchrotron radiation sources, demanding large-area high-speed detectors. The speed, convenience and efficiency of detectors based on a CCD chip coupled to a primary conversion phosphor *via* a fibre optic taper have led to such systems being currently the most widely adopted at macromolecular crystallography beamlines (Tate *et al.*, 2006 ▸; Minor *et al.*, 2000 ▸). We use the term ‘phosphor-taper-CCD detector’ (hereinafter referred to as p-t-CCD detector) to distinguish between this type of device and other X-ray detectors based on CCD technology, such as those coupled to lenses or other optical components (Gruner *et al.*, 2002 ▸) and direct-detection CCDs (Clarke, 1994 ▸). Recent developments in detector technology have led to large photon-counting hybrid pixel-array detectors suitable for macromolecular crystallography that offer improved speed and noise characteristics over current p-t-CCD detectors. Nevertheless, a large amount of data has already been accumulated using p-t-CCD detectors and it is likely to be some years before such detectors are superseded in macromolecular crystallography. Indeed, integrating detectors retain an advantage over photon-counting devices in situations where beam flux is very high, such as during the intense pulse of a free-electron laser source, owing to the finite dead time of counting detectors.

Integration software for macromolecular crystallography evolved in tandem with the technology used to perform the experiments, particularly detector technology. The underlying experimental method essentially remained the rotation method of Arndt & Wonacott (1977 ▸), with the data collected as multiple frames or images by two-dimensional area detectors. Radical redesign and overhaul of the software has not been required; rather, existing algorithms have been adapted to cope with new detector types and improvements in methodology, such as profile fitting, auto-indexing and handling of fine-sliced oscillations. For example, the first versions of *MOSFLM* (Leslie, 1992 ▸) were written to handle data recorded on X-ray film and, later, image plates. With the widespread uptake of p-t-CCD detectors at synchrotron sources, the existing integration procedures were updated for the new type of images produced (Leslie, 1999 ▸, 2006 ▸). However, assumptions about pixel correlations and instrumental errors appropriate for earlier detectors are not strictly valid for current and future detectors. Here, we investigate and suggest improvements in error estimation that can be made over adaptations of legacy procedures by reformulating models for integration specifically for data recorded on a p-t-CCD area detector.

### 1.1. The importance of measurement error estimates   

The errors associated with integrated diffraction-spot intensities are a combination of the inherent random sampling expected from counting statistics with the instrumental response and experimental errors. The instrumental and experimental errors are either random, such as detector read-out noise, dark signal, and variations in the dose per exposure caused by factors such as beam instabilities and shutter jitter, or systematic, caused by absorption, crystal decay, nonuniform response of the detector, inaccurate distortion correction, detector gain or other factors. The distinction between systematic and random error is not always clear. Here, we refer to the definitions provided by Bevington & Robinson (1992 ▸). Briefly, in any experiment, if a particular measurement is made many times, random errors are those that affect the precision of the mean value of those measurements, yet with enough measurements the effect of these fluctuations is overcome and the sample mean is a good estimate of the true value. In contrast, systematic errors are reproducible discrepancies of measurements from the true value, such that the mean of a sample of many measurements is not an accurate estimate of this value.

The accurate estimation of errors is of great importance throughout the process of crystallographic structure solution (Borek *et al.*, 2003 ▸). Generally, errors are used as weights to indicate the reliability of each measurement. In current practice, it is typical to use weights obtained from the estimated errors of individual measurements during the averaging, or merging, procedure to obtain the best estimate of a Bragg spot intensity, and consequently its underlying structure factor, from multiple observations. Subsequent use of the value of a merged structure factor implies the acceptance of a certain error model, because its magnitude is a function of the relative weights of its contributing observations.

New methods are now emerging for the treatment and exploitation of unmerged data. For example, it has been demonstrated that phasing power can be increased by dose-dependent modelling of site-specific structural changes due to radiation damage when unmerged structure factors are used (Schiltz *et al.*, 2004 ▸). In addition, the phenomenon of anisotropic anomalous scattering of polarized synchrotron radiation also provides a powerful source of phase information by breaking symmetry equivalence, an effect which is clearly lost if data from symmetry mates are merged (Schiltz & Bricogne, 2008 ▸). These examples demonstrate how apparently deleterious effects can actually be put to beneficial use by remodelling of the experiment to account for physical processes in more detail. Under these circumstances, each unique observation must be presented with its own accurate estimate of error.

The success of structure determination depends critically on the magnitude of the errors, particularly for experimental phasing procedures that require accurate values for small differences between measured intensities (Borek *et al.*, 2003 ▸). Not only does the accurate estimation of errors allow determination of the quality of a marginal signal, but the error estimates themselves are incorporated into the procedures for model fitting and estimation. The introduction of Bayesian inference techniques to macromolecular crystallography has very successfully reformulated the problem of deriving knowledge from data in terms of maximizing the probability of observing those data given parameterized hypotheses (maximum likelihood) and considering the probability of those hypotheses in the light of prior knowledge (full Bayesian estimation) (Bricogne, 1997 ▸). These techniques now permeate most areas of modern macromolecular crystallography. Likelihood targets constructed for tasks such as experimental phasing (de La Fortelle & Bricogne, 1997 ▸; McCoy *et al.*, 2004 ▸, 2007 ▸), molecular replacement (Read, 2001 ▸; McCoy *et al.*, 2007 ▸) and structural model refinement (Murshudov *et al.*, 1997 ▸; Blanc *et al.*, 2004 ▸) are probability distributions, with variances that combine errors in the calculated (model) values with errors in the observed values. Measurement errors are thus naturally construed not merely as weights but as limiting factors in the degree of belief held about derived knowledge.

### 1.2. Currently employed models for error estimation   

The number of photons forming a diffraction spot at the detector face is well described by a random variable that follows a Poisson distribution, such that if the spot is composed of *N* X-rays, the best estimate of the standard deviation of the underlying distribution is given by *N* ^1/2^. For an ideal detector, the uncertainty in the data is determined entirely by the unavoidable statistical fluctuations in the incident photon flux. Such a detector has a detective quantum efficiency DQE = 1, where DQE is defined as the ratio of the squared signal-to-noise ratio at the output of the detector to the squared signal-to-noise ratio at its input. 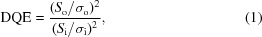
where *S* refers to the signal, σ to the noise, and the subscripts o and i to output and input, respectively. Real detectors never achieve the limit of perfect DQE. Integrating detectors such as p-t-CCD devices collect a signal that is proportional to the number of incident photons during an exposure time. Stochastic processes occurring as part of the detector physics lead to a variation in response per event and therefore degrade the output signal-to-noise ratio. Calculations of the DQE for Bragg-spot integration on a particular p-t-CCD detector have shown it to vary from ∼0.35 for a weak spot (500 X-ray photons) to ∼0.7 for a spot with 10^4^ X-ray photons (Phillips *et al.*, 2000 ▸). Despite this, it is still common to assume that, at the point of integration, the detector response follows Poisson statistics, *i.e.* to assume a perfect photon-counting detector. It is also assumed that not only is the recorded signal Poisson distributed, but that each pixel contributing to that signal is independently Poisson distributed, without correlations with neighbouring pixels. In fact, for p-t-CCD detectors the combined point-spread function (PSF) of the phosphor and optical chain results in a reduction of the optical resolution of the detector and introduces correlations between pixels that may extend many neighbours deep. The software correction of geometric image distortions introduced by the fibre-optic taper (FOT) also increases local correlations, by spreading the signal at each pixel position between a cluster of pixels in the final corrected image. One way to deal with deviation from the assumptions of ideality is to inflate the error estimates using an additional term to address measurement uncertainty introduced by the detector. *MOSFLM* incorporates an instrumental error term to account for a particular type of error dependent on the intensity and spot shape expected if measurements are performed by X-ray film densitometry. Although the physical justification for this term is not appropriate for p-t-CCD detectors, the resulting estimates are reportedly more realistic, particularly for strong reflections for which the random errors are a smaller proportion of the total error, such that the systematic errors dominate (Leslie, 1999 ▸). One source of systematic error is the nonuniformity of signals recorded at different regions of the detector. This nonuniformity increases the scatter of intensities between crystallographically related reflections that are to be merged together. As discussed in §[Sec sec6.3]6.3, the size of this error depends on the sharpness of the diffraction spot, *i.e.* the systematic errors are worse for stronger smaller spots. Other sources of variation between crystallographic symmetry-related reflections due to apparatus and experimental imperfections may also be partly accounted for by the instrumental error term. Unfortunately, application of such an error-inflating factor at the point of integration masks the distinction between the measurement uncertainty of individual reflections and the additional error inflation required to meet the observed scatter when a scaling model is applied, *e.g.* by *SCALA* (Evans, 2006 ▸).

### 1.3. Motivation and outline of the current work   

It would be desirable for the initial error estimates made during integration to reflect accurately the true random error in each individual measurement of an integrated Bragg spot. This part of the error is a combination of the underlying Poisson statistics of the X-ray source with the instrumental response, which includes physical factors such as read-out noise and factors related to processing of the images, for example the distortion correction, which increases neighbouring pixel correlations. It follows that, in order to obtain accurate estimates of the total random error in a single measurement, it is essential to have an accurate model of the detector response.

Current methods of error estimation rely on the comparison of the observed scatter with the expected error in the data through methods such as normal probability analysis (Evans, 2006 ▸). This leads to an estimate of the total error in each merged intensity that includes all random errors and all residual systematic errors not removed by scaling. With accurate knowledge of the measurement uncertainty described above, it may be possible to isolate and quantify these residual sources of systematic error and any additional random error in the data. We hope that breaking down the total error into its individual components will lead to a quantitative assessment of all hardware, experimental factors and sample-related effects (such as radiation damage) that influence data quality in macromolecular crystallography.

In the text that follows, we describe in detail a computer simulation of a p-t-CCD detector, and then its statistical response is investigated by modelling the detector as a cascade of events through a series of gain stages. The signal and associated uncertainty are first considered generically for values recorded in a region of interest without assessment of a background level. This formulation is used to derive the DQE of the simulated detector and it is then adapted for use in a summation integration procedure, where the signal is given by a localized Bragg-spot intensity with a subtracted X-ray background. We then describe a series of simulations and experiments to investigate the application of this noise model to the simulated detector and how it compares with a real p-t-CCD detector. It is made clear that, for profile-fitting error estimates, the model must be extended to take into account the effects of pixel value correlations, and indeed that these correlations have a nonuniform structure caused by the distortion correction. We envisage that the presented model, and its future extension to profile-fitting error estimation, may be applied to real p-t-CCD detectors within integration software to provide realistic best estimates of the true random measurement errors for each integrated intensity. We present ideas for improvements to integration routines, with particular reference to the *MOSFLM* program. However, the model for a typical p-t-CCD detector is generally applicable, so other integration packages may benefit equally.

## 2. p-t-CCD area detector simulation   

In order to investigate ways in which errors introduced by the detection process could be more accurately modelled, a simulation of a p-t-CCD detector module of 1024 × 1024 pixels was produced as a package for the statistical programming environment R (R Development Core Team, 2009 ▸). This simulation consists of a set of functions that model components of the detector, such as the phosphor screen, FOT and CCD chip. As well as addressing the physical processes that result in a raw image, the simulation also includes corrections that are applied to the image to account for dark signal, geometric distortion by the FOT and response nonuniformity. In addition to the functions simulating detector operation, the package includes functions for summation integration and profile-fitting integration, as well as functions to read images produced by both ADSC (Area Detector Systems Corporation) and Rayonix p-t-CCD detectors, and reflection data files in CCP4 MTZ format (Collaborative Computational Project, Number 4, 1994 ▸). This enables convenient manipulation of images and reflection data within the R environment, with access to all of the statistical and graphical capabilities that entails. The code makes use of the general polygon clipping library for R, *gpclib* (Peng *et al.*, 2010 ▸), based on the University of Manchester GPC library, and multivariate normal random deviate generation by the *mvtnorm* package (Genz *et al.*, 2010 ▸). All the functions presented here are collected into a single R package called *DISP*, standing for diffraction image statistics package, which can be obtained without charge from the authors. R is freely available under the terms of the GNU General Public Licence (GPL), Version 2.

Use of the simulation involves the application of a series of functions to convert a table of X-ray photon positions incident on the detector face into a matrix of 16-bit integer values corresponding to the pixel values of an image. These functions simulate the real-life chain of events involved in detection, starting with X-ray absorption in the phosphor, followed by amplification to a shower of light photons, and transmission through the FOT and other couplings to the sensor to form stored charge. Read-out of the simulated detector returns a raw image with pixel values corresponding to digitization of the stored charge, subject to read-out noise and dark signal for the length of the exposure. If a flat-field image and a calibration array for the distortion correction have been calculated for the detector, this raw image can be corrected, producing an image that mimics the usual output seen by a crystallographer performing a data collection. The correction procedures used by *DISP* are not exact algorithmic reproductions of published procedures for real detectors but are implementations of the same ideas, intended to produce images with features closely related to real images. In particular, exact knowledge of the taper distortion made it easier to calculate the distortion correction table, rather than following the empirical method required for real detectors (Stanton *et al.*, 1992*a* ▸; Paciorek *et al.*, 1999 ▸; Barna *et al.*, 1999 ▸). Nevertheless, the important feature of distortion correction is preserved – the distribution of pixel values between a cluster of neighbouring pixels in the corrected image. The main steps taken to produce the simulated images are summarized in the following subsections.

### 2.1. Amplification at the phosphor screen   

The simulated X-ray source was assumed to be monoenergetic at 12 keV and X-rays were assumed to arrive perpendicular to the detector face. These assumptions are sufficient for current purposes. However, the response statistics of a real p-t-CCD detector are affected by X-ray energy and obliquity of incidence. This should be taken into account in a future implementation of a complete model for integration of real data. The phosphor screen was assumed to have a uniform response. Rather than explicitly model the detailed physical mechanism of X-ray interaction with the phosphor, we used precalculated results from a Monte Carlo simulation (Liaparinos, 2009 ▸; Liaparinos *et al.*, 2006 ▸; Liaparinos & Kandarakis, 2009 ▸) to model both the PSF and the phosphor light-yield distribution, for light photons that escape the back surface of the screen towards the downstream detector components (*i.e.* transmission mode). The light-yield distribution is also referred to as the phosphor scintillation spectrum (Mickish & Beutel, 1990 ▸; Beutel *et al.*, 1993 ▸), or elsewhere as the pulse-height spectrum (Liaparinos & Kandarakis, 2009 ▸). The form of this distribution determines the information factor or Swank noise for the phosphor, which gives the contribution of variable light output to the overall system DQE (Swank, 1973 ▸; Beutel *et al.*, 1993 ▸).

**Figure 1:**
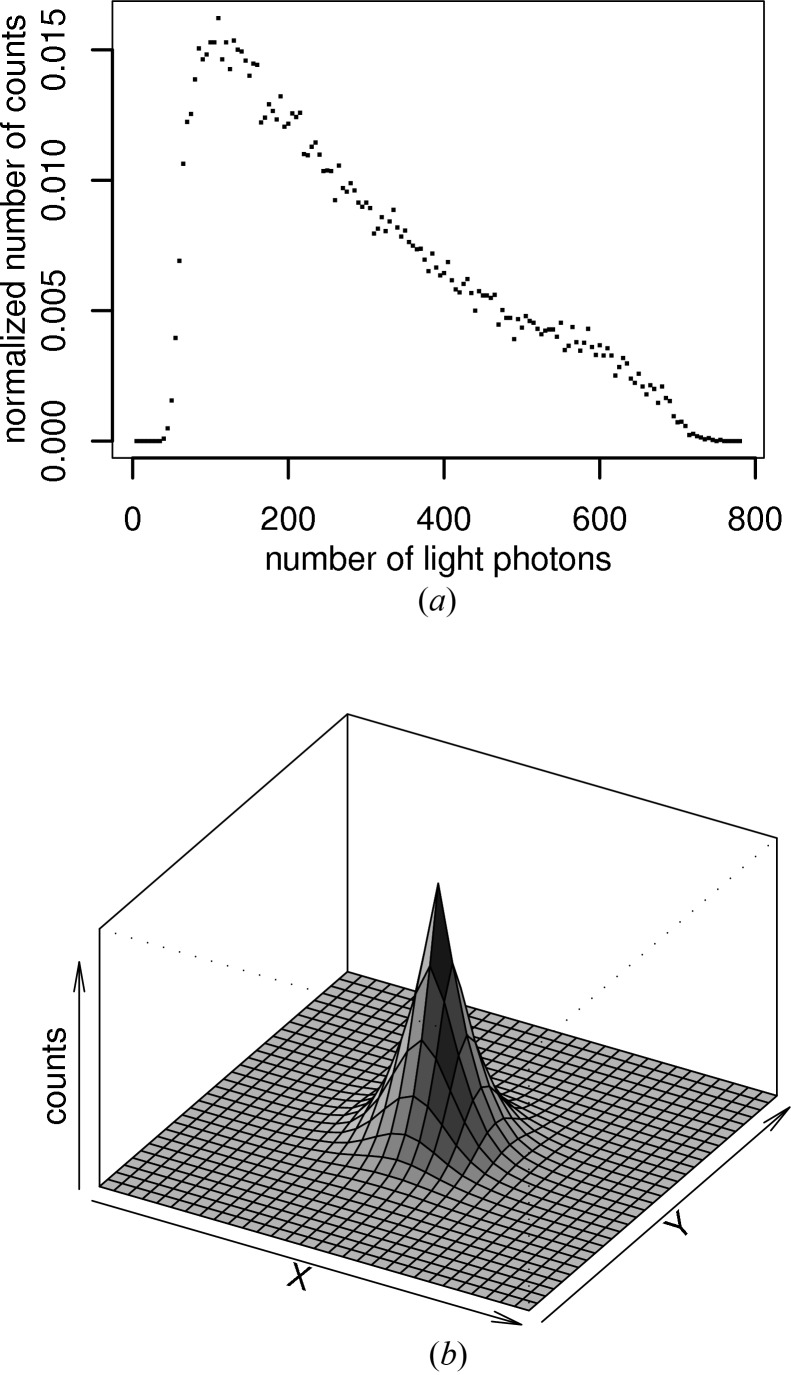
Phosphor simulation details. (*a*) The light-yield or scintillation spectrum for the simulated phosphor, with parameters as given in Table 1 ▸. Although the mean number of light photons emitted per 12 keV X-ray is 281.5 photons, the distribution is rather broad, so events with much higher or lower amplification gains are common. (*b*) The point-spread data were determined by simulating a pencil beam of X-rays incident on the phosphor and binning output counts into a pixel grid with a 10 µm pitch positioned immediately behind the phosphor screen. A surface was then fitted over the counts. Note that this graph represents the point spread of the phosphor alone.

The PSF of a real phosphor typically displays a sharp peak, accompanied by long tails caused by scattering of light. Data for the mean PSF of complete detector systems have been reported fitting an exponential function (Westbrook & Naday, 1997 ▸), and recently a model was proposed for the PSF of a phosphor for the diffraction-image simulator *MLFSOM* by considering the geometry of a point source positioned above the pixel plane (Holton, 2008 ▸). By avoiding an explicit model and simply sampling a precalculated PSF, the results produced by *DISP* are realistic and require minimal computational expense. It should be noted that the PSF data have been modified from the originally supplied data in order to produce a radial profile of smoothly interpolated values suitable for sampling. The full-system PSF is also inflated slightly compared with an exact transmission of the phosphor PSF, in order to produce realistic values that account for other elements in the optical chain. Hence, the fine details of the PSF used in *DISP* should not be considered indicative of the accuracy of the original phosphor simulation (Liaparinos *et al.*, 2006 ▸). Details of the phosphor model are given in Table 1 ▸ and Fig. 1 ▸.

### 2.2. Image transmission by the fibre optic taper   

*DISP* uses a simple model of an FOT, to account only for demagnification and continuous distortions. Nonuniformities, such as ‘chicken-wire’ patterns caused by bundling of optical fibres or shear distortions that inflict real tapers (Coleman, 1985 ▸), were not modelled. The image distortion introduced by the FOT was modelled as a radial function, so that a symmetrical pincushion or barrel-type distortion can be easily produced, although real tapers usually show more complicated patterns of distortion, breaking the radial symmetry. The uniform losses of the simulated optical chain lead to an overall transmittance of 4.9%, using realistic parameters for a taper with a demagnification ratio of 2.7:1, as detailed in Table 2 ▸.

### 2.3. Charge accumulation and CCD read-out   

The CCD quantum efficiency was assumed to be 35%, the remainder of the incident photons being lost by reflection or absorption in gates. For those photons that do interact, a unity photon-to-electron conversion gain was assumed. These photons were binned into the appropriate pixels according to the position at which they exited the taper, with each pixel forming a 30 µm square on the sensor (similar to real CCD sensors with a 2 × 2 hardware binning mode). Dark signal was accumulated during exposure at a rate of 0.01 electrons per pixel per second. Read-out noise was modelled by generation of a random deviate for each pixel, taken from a normal distribution with a mean of zero and a standard deviation of ten electrons, which was then added to the pixel electron charge. For read-out, an analogue-to-digital converter (ADC) conversion rate of five electrons per analogue-to-digital unit (ADU) was used over the whole scale. No near-full-well nonlinearity was modelled. A constant offset bias of 500 ADU was added to the pixel values. Real CCD sensors have arbitrary ADC bias voltages that vary for different read-out channels. Pixel values were capped at the 16-bit integer maximum, but no pixel bleeding effects have been considered for overloaded pixels. The digitized images produced by this stage are referred to as raw images. As with real detectors, whole-image corrections for nonuniformity and distortion were then performed, to produce the corrected images that are used by the most common data-integration procedures.

### 2.4. Dark subtraction and nonuniformity correction   

The order in which corrections were applied for *DISP* was chosen to be the same as for the Rayonix series detectors (Doyle, 2006 ▸). The first stage in raw-image correction is to subtract a dark image, which also removes the bias offset. Subsequent correction procedures involve multiplicative arithmetic on pixel values, which would result in incorrect values if performed with the bias present. In the simulation, the phenomenon of ‘zingers’ (spots arising from the direct impact of X-rays, cosmic rays or radioactive decay events on the CCD sensor) was not modelled and all images were assumed to accumulate dark signal for 1 s. A single dark image was generated for correction of all raw images. Following dark subtraction, raw images were corrected for nonuniformity using a flat-field response image. Because the simulated detector has an inherently uniform response (apart from the image-density variation introduced by the spatial distortion discussed below), as a correction the procedure is superfluous. Nevertheless, it has an effect on data quality, and therefore we chose to model it. The uniformity of response of the simulated detector ensures that systematic errors due to sharp features in a source signal that are unlike the flood field used for calibration, as discussed in §[Sec sec6.3]6.3, are not present. However, there are still systematic errors present because of imprecision in the flood field, *i.e.* the random difference between the recorded flood field and the true, uniform, response. Performing the nonuniformity correction for the simulated detector is therefore akin to introducing a systematic error into the otherwise uniform response.

To perform the nonuniformity correction, each pixel in a dark-corrected raw image was divided by a value stored at the equivalent position in the flat-field normalizing array. The flat-field array was calculated in advance by the average of a set of dark-corrected flood-field images, which had been processed to remove known deviations from flatness in the flood-field signal. The luxury of simulation allows an ideal uniform flood field to be supplied to the detector face, which is not usually practical in reality. However, even in this case one source of deviation from flatness in the recorded signal remains. This residual error is due to the FOT, because the distortion it causes is non-area preserving, leading to systematic differences in the photon density across the image. This means that before the averaged flood-field image could be made into a flat-field image for use in the nonuniformity correction, it had to be scaled according to the effective collection area of each CCD pixel when it is mapped back along the FOT to the detector face. The resulting flat field was then normalized, so that the values represented the scale factor by which the equivalent pixel in the image under correction is greater or less than the expected uniform value.

### 2.5. Distortion correction   

Correction of the image distortion was achieved by re­apportioning pixel values according to the overlap of the raw-image pixel grid with a new noncommensurate nonorthogonal grid that takes into account the spatial distortion in the original image (Paciorek *et al.*, 1999 ▸). In *DISP* this was achieved by mapping the CCD pixel vertices back along the FOT to form quadrilaterals at the detector face. Overlaps between this grid of quadrilaterals and an orthogonal grid of pixels defined at the detector face with a 73 µm pitch were calculated, and the pixel values were then distributed into the orthogonal grid according to these overlaps. In this simulation, a simple radially symmetric model of fibre-optic distortion was used, based on a third-order polynomial, with none of the local discontinuities or other imperfections often present in real tapers. Because the form of distortion was known exactly, it was not necessary to simulate the usual procedure of exposure through a grid mask to measure the distortion and interpolate for all pixels (Stanton *et al.*, 1992*a* ▸; Barna *et al.*, 1999 ▸).

**Figure 2:**
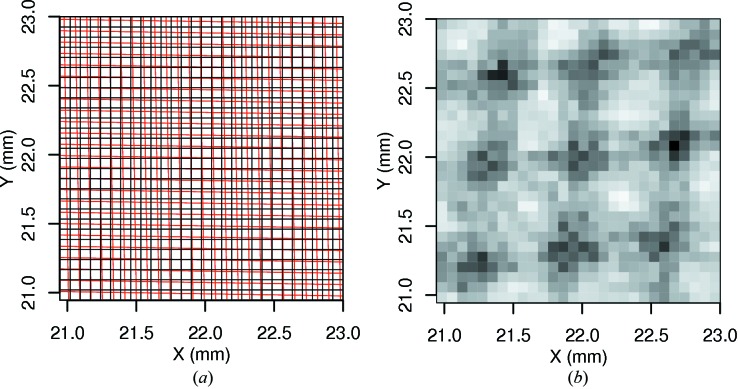
Distortion correction. (*a*) The orthogonal grid of pixels for a 2 × 2 mm area of the simulated detector face (black), superimposed over the grid of CCD pixels (red), translated back along the taper to show their equivalent areas on the detector face. (*b*) 100 flood images with an expected value of 100 X-ray photons per pixel were simulated, with full corrections. This image shows the variance of the pixel values calculated over the image ensemble in the same region of the detector as the image in (*a*). It is evident that the variance is higher (darker pixels) in regions where the pixel grids on the top panel match well, whereas in regions where they match poorly there is a smoothing effect, leading to lower variance but higher covariance.

The effect of the distortion correction was visible in corrected images as a nonuniform smoothing, resulting in Moiré-like patterns. In areas where the original and distortion-corrected grids closely matched one another, little intensity was shared between pixel neighbours and covariance between these neighbours remained low. In other regions where the grids matched badly, the intensity of a pixel may have been spread between four or even more neighbouring pixels in the corrected image, increasing the covariances between pixels and their neighbours and producing a visible smoothing. Although this effect is visible on individual corrected frames, it is especially apparent on an image of the variance at each pixel calculated for an image ensemble, as shown by Fig. 2 ▸. This nonuniformity of the local covariance structure has a direct effect on error estimation for profile-fitting integration, as discussed in §[Sec sec6.2]6.2.

## 3. Theory: detector response   

### 3.1. Non-Poisson detector statistics   

A detection event is naturally described by a chain of processes, with the output at one stage feeding into the input of the next. Each stage has a particular gain, or loss, that is subject to a probability distribution. A detector model of this type was proposed by Breitenberger (1955 ▸) and has been applied for the calculation of zero-frequency DQE for various detectors, including area detectors used in diffraction studies (Zweig, 1965 ▸; Arndt & Gilmore, 1979 ▸; Stanton *et al.*, 1992*b* ▸). For more general imaging tasks, a calculation of spatial frequency-dependent DQE may be more appropriate (Cunningham, 2000 ▸), such as that of Williams *et al.* (1999 ▸) for a p-t-CCD-type detector. The error response of the detector chain can be modelled by considering the relative variance of the output signal, a dimensionless quantity defined as the variance divided by the squared signal. The overall relative variance can be broken down into the sum of the relative variances normalized by the number of input quanta at each stage in the cascade (Breitenberger, 1955 ▸). The stochastic stages forming the simple detector model for the simulation reported here are as follows: X-ray incidence on the phosphor screen, absorption of a fraction of those X-rays, amplification due to emission of light photons from the phosphor, transmission of light photons through the FOT and other optical couplings to the CCD, and conversion of light photons to electron–hole pairs on the chip.

The event cascade, prior to read-out and digitization, which add extra noise, can be described by means of an overall signal 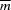 in units of electrons stored in the pixels of the CCD sensor. This can be broken down into the product of the number of incident X-ray photons 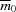 with the phosphor quantum absorption 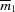, the quantum amplification 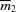 for the production of light photons, the overall fraction 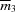 of light photons transmitted to and absorbed by the CCD, and 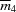 for the quantum yield of electrons on the chip, 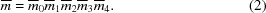
We are interested in the signal and error integrated in a small region of interest on the detector, defined by the integration measuring box surrounding a single Bragg spot. The number of X-ray photons incident in the region of interest on the screen is a Poisson-distributed variable, with mean and variance 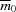. The relative variance in this quantity is therefore given by *R*_0_ = 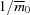. The proportion of these X-rays that interact with the screen is described by the phosphor quantum absorption (Swank, 1973 ▸). This is modelled by a binomial distribution (Breitenberger, 1955 ▸), with the interaction probability or fractional gain 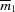 equal to the quantum absorption, with variance 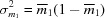. The relative variance for this stage is therefore *R*_1_ = 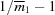. The third stage concerns the phosphor amplification gain, described by the scintillation spectrum of the phosphor material, with mean value 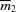, variance 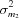 and thus a relative variance given by 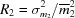. The fourth stage in the chain describes the absorption and other losses of light through the optical couplings to the CCD pixel. This is also modelled as a bi­nomial distribution (Arndt & Gilmore, 1979 ▸), with transmission 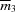 and variance 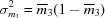, giving the relative variance for this stage *R*_3_ = 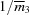 − 1. The simple model employed by the simulation assumes monoenergetic emission by the phosphor at 545 nm. Photons with this energy may create no more than one electron–hole pair (Westbrook & Naday, 1997 ▸). The probability of interaction has already been incorporated in the transmission 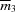, so a single photoelectron will always be produced for each light photon, giving 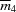 = 1 with a variance of zero. The total relative variance of the quantity 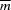 is therefore 
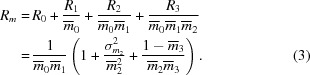



The relative variance of the number of electrons stored in pixels in the region of interest on the chip is converted into the actual variance in this quantity by multiplying by the squared output signal for the combined cascade stages, 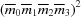, 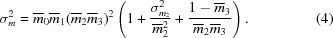



The cascade chain is responsible for the number of electrons 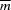 stored on the chip in the region of interest. However, the signal we actually have access to is a different quantity, 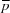, which is subject to further error due to the CCD read step and conversion from analogue voltage to digital units. For a particular number of electrons *m* stored in a region of interest consisting of *N* raw-image pixels, the sum of pixel values *p* may be calculated by 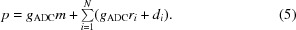
where *g*_ADC_ is the analogue-to-digital converter gain between electrons and ADU, *r*_*i*_ is the read-out noise in electrons at pixel *i*, *d*_*i*_ is the digitization error in ADU at pixel *i*, and *N* is the number of pixels over which the signal is collected. Real CCDs often have multiple read-out channels, each with their own ADC gain. In this simulation, *g*_ADC_ is assumed to be a fixed known quantity that is equal for each pixel and contributes no variance. The read-out noise is drawn from a normal distribution with a mean of zero and a standard deviation of σ_*r*_ electrons. Digitization noise is strongly signal-dependent and difficult to analyse directly. Nevertheless, in favourable cases, such as for CCDs where the read-out noise has a magnitude greater than 1 ADU, it is possible to employ the pseudo-quantization noise model as a good approximation. In this model, the digitization error is sampled from a uniform distribution with a range of 1 ADU (Widrow *et al.*, 1996 ▸). The expected value of the signal can therefore be expressed as 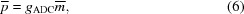
with the noise in the signal given by 
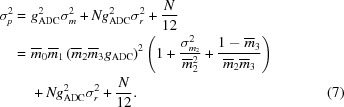



The apparent gain of the detector considering only absorbed photons is given by *G* = 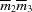 *g*_ADC_. This definition matches that of *MOSFLM* and is useful when making error estimates from pixel values, which clearly can only consist of counts from detected X-rays. However, it should be noted that the independently measurable gain is rather *G*′ = 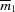 *G*, and it is this that should be considered for calculations of the DQE (Ponchut, 2006 ▸). Using the definition of *G* given, the above expressions can be written as 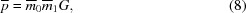


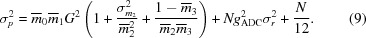



For a general detector where the parameters are not all known individually, we may fold the terms into a ‘cascade factor’ γ and ‘pixel factor’ ψ, 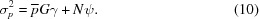
Usually, the signal is estimated from a single sample measurement. If a particular single image contains a region in which the sum of pixel values is *p*, this can be used to estimate the error 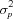. Strictly speaking, this assumes a normal distribution of each pixel value, so that the mean coincides with the most probable value, but this is generally a good assumption. Thus, 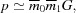
and therefore 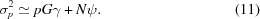
For the described simulation, values for the relevant quantities in the above expression (assuming monoenergetic photons at 12 keV) are given in Table 2 ▸.

For comparison, it is convenient to consider the signal in units of detected X-ray photon counts rather than ADU, to compare the variance with the expected Poisson variance of a photon counter given the same signal. If the expected value of the signal in detected X-ray photon counts 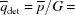 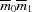, then the variance is given by 
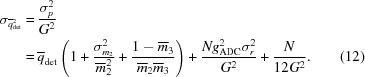
Substituting the values from Table 2 ▸ into this expression gives 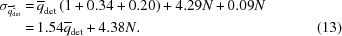
From this expression, it can be seen that the simulated detection cascade performs significantly worse than an exact photon counter with the same quantum absorption, with a cascade factor γ = 1.54. In addition, the read-out noise and digitization error contribute a small but significant extra term to the variance, depending on the number of pixels considered in collecting the signal.

### 3.2. Detective quantum efficiency   

**Figure 3:**
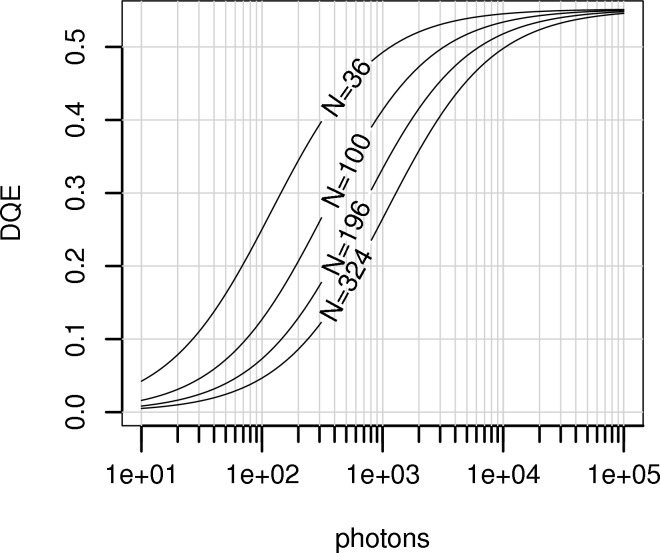
Zero-frequency DQE, taking into account the detector cascade and pixel noise. The family of curves shown gives the DQE for the simulated detector as derived by equation (14) for regions of interest consisting of 6 × 6, 10 × 10, 14 × 14 and 18 × 18 pixels. For relatively weak signals of less than 1 × 10^4^ X-ray photons, the size of the region of interest becomes important owing to the pixel-noise component of the DQE expression. At high flux, the DQE is limited by the phosphor quantum absorption and the response of the cascade chain.

Considering the cascade model and pixel noise, the statistical quality of the detector response can be summarized by calculating the zero-frequency DQE (Westbrook & Naday, 1997 ▸; Arndt & Gilmore, 1979 ▸; Stanton *et al.*, 1992*b* ▸). If the incident signal is governed by Poisson statistics, with mean and variance given by 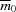, then from equations (1), (8) and (10) the DQE may be written 
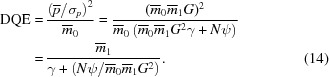
It is immediately clear that the DQE is limited by the phosphor quantum absorption 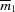 and further degraded by the response fluctuations due to the cascade chain, summarized by γ. For the simulated detector presented here, this sets an upper limit for the DQE in the absence of pixel noise of 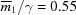. The read-out and digitization noise further degrade the DQE according to the number of pixels *N* over which signal is recorded, especially for weak signals with a relatively low incident flux 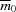. To illustrate this, the theoretical DQE for the simulated detector is plotted in Fig. 3 ▸.

### 3.3. An improved error model for summation integration   

The expression for detector error response given by equation (11) can also be used to improve a standard algorithm for summation integration error estimation, by including determination of the X-ray background. We do not consider systematic errors such as those due to inaccurate nonuniformity correction, discussed further in §[Sec sec6.3]6.3, or include an instrumental error factor that partially accounts for such errors. Systematic errors in the detector response are best identified explicitly by a procedure for merging and scaling data. At this stage, we envisage assignment of an accurate measurement error estimate for the random error of each measured intensity, based upon knowledge of parameters describing the detector response. The parameters, given for the simulation in Table 2 ▸, are fixed properties of the detector, apart from *N*, the number of pixels in the raw image over which the signal is measured. The use of corrected images for integration affects the handling of read-out and digitization noise. Distortion correction procedures usually ensure that the total number of pixels in the corrected image is the same as in the raw image. However, a fraction of the raw image pixels are discarded by the correction, as these are located in the unexposed region around the edges of each CCD sensor, outside the taper–chip interface. Therefore, the total number of raw-image pixels contributing read-out and digitization noise is slightly less than the total number of pixels in the corrected image. For any particular measurement box defined on a corrected image, the number of contributing raw pixels, *N*, could in principle be calculated from the distortion map. However, it may be more practical to measure the total read-out and digitization noise directly, using the variance at each pixel position over a set of dark images that have had full corrections applied, or simply to approximate *N* by the number of pixels in the corrected image measurement box.

**Figure 4:**
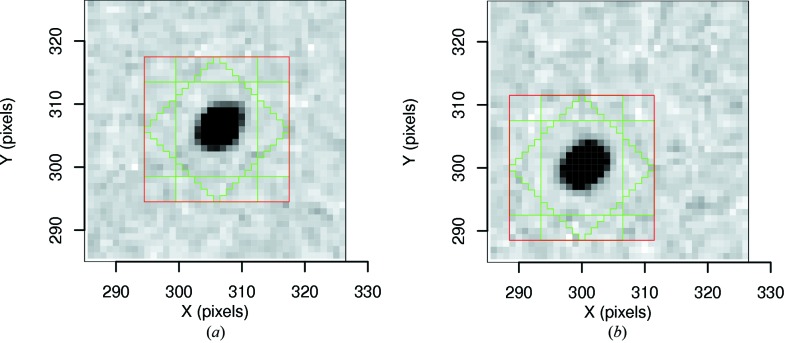
Measurement boxes with parameters *NX* = *NY* = 23, *NRX* = 5, *NRY* = 4 and *NC* = 11 used for the integration of spots on corrected images as presented in Tables 3 ▸ and 4 ▸, displayed over an image from each ensemble of corrected images. (*a*) In zone *A*, the measurement box central pixel is *X* = *Y* = 306 pixels. (*b*) In zone *B*, the centroid of the spot has been shifted in both *X* and *Y* by six pixels, such that the centre of the measurement box is at *X* = *Y* = 300 pixels.

Expressions for the summation integrated intensity and estimated error were derived by Leslie (1999 ▸). The treatment here is similar, but avoids the assumptions that each pixel is an independently distributed Poisson source. First, a measurement box is formed around the spot, described by the same integer parameters as used in *MOSFLM* (Leslie, 1999 ▸). That is, *NX* and *NY* define the horizontal and vertical side lengths in pixels and are both odd integers, to ensure a central pixel in the measurement box. *NRX* and *NRY* give the rim widths in pixels separating the background and peak regions. Finally, *NC* is a corner cut-off parameter. Examples of measurement-box definitions are shown in Fig. 4 ▸. In the measurement box, pixels have the pedestal offset for corrected images subtracted from them. This constitutes a noiseless bias in the background. As the background will be subtracted, this bias will not affect the integrated intensity. However, removal of this offset is important when estimating error, as in that case background counts are included, leading to a systematic bias in the error estimate unless the offset is removed. The size of this bias is dependent on the particular detector system’s chosen offset level. *MOSFLM* has a keyword, ADCOFFSET, that may be used to set the right pedestal level, but it is not clear if this is always correctly exploited by users.

The background level is determined as described by Rossmann (1979 ▸), by an unweighted least-squares fit of a plane to the background region pixels. The integrated intensity (in ADU) is given by 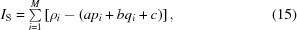
where the summation runs over *M* peak-region pixels, with intensities ρ_*i*_ and coordinates (*p*_*i*_, *q*_*i*_). The background plane parameters *a*, *b* and *c*, determined outside the peak region, are used to interpolate background values inside the peak.

Rather than estimating the error in the background term from the quality of fit of the background plane, a simplification can be used that avoids necessitating knowledge of the correlations between pixels. The measurement-box parameters stipulate that the measurement box has *mm* symmetry. Thus, the intensity of the pixels constituting the background under the peak can be given by the product of the number of pixels in the peak region and the average background pixel value, 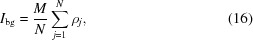
where the sum is over the *N* background-region pixels. This simplification allows the estimated variance of *I*_bg_ to be addressed in terms of the total signal in the background region, considering the cascade and pixel noise model, by comparison with equation (11): 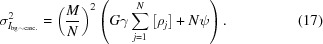
For the peak region, the error estimate for the total intensity is taken directly from equation (11). Combining the peak and background parts gives a complete expression for the estimated error in the summation integrated intensity, taking into account its distribution according to the cascade model with pixel noise: 
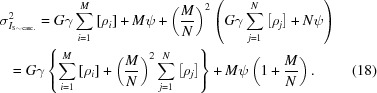
The absorbed photon gain *G* and the cascade factor γ are dependent on both the detector characteristics and the X-ray energy. It would be important to have reasonable values for these quantities available at the point of integration, in order to produce realistic error estimates. Sometimes an estimate of *G* is made using the variance-to-mean ratio in a region of interest of a single flood image, where the variance is calculated as a spatial fluctuation. This calculation is based on the assumptions that each pixel is independently distributed and follows Poisson statistics. As neither assumption is true for a p-t-CCD detector, this method should be avoided.

## 4. Methods: simulations and experiments   

### 4.1. Integration of simulated spot image ensembles   

The simulation was operated in order to produce an ensemble of 1 × 10^5^ raw images containing a single spot (in zone *A*) with a two-dimensional Gaussian profile on a uniform flood-field background. A second ensemble of 1 × 10^5^ raw images was generated with the spot centroid shifted by six pixels compared with the first set, in order to sample a different region of the detector (zone *B*). The number of X-ray photons constituting each spot was selected from a Poisson distribution with mean 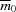 = 1 × 10^4^. A further two image ensembles were formed by applying full corrections to the two sets of raw images. Suitable measurement boxes were found in all cases (see Fig. 4 ▸ for examples of corrected images of spots from both zones), which allowed recovery of the expected intensity, *i.e.* 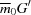 = 8206 ADU. The flux of the random uniformly distributed X-ray background was chosen to give a mean value of 20 X-ray photons per pixel region, as defined at the detector face.

#### 4.1.1. Summation integration   

Summation integration was performed on all images using a routine written for *DISP* based on the algorithms in *MOSFLM* (Leslie, 1999 ▸). Errors were estimated using both the *MOSFLM* formulation and the new form given by equation (18). In both cases, the value for the absorbed photon gain was calculated from G = 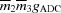.

#### 4.1.2. Profile fitting   

Integration by profile fitting was also performed using the same measurement-box parameters and a profile-fitting routine based on that of *MOSFLM* (Leslie, 1999 ▸). Profiles were formed by taking the mean of the bias- and background-corrected measurement boxes for additional sets of 20 raw and corrected images of spots recorded in zones *A* and *B*, and rounding pixel values to the nearest ADU. Error estimations (described in detail in Appendix *A* [App appa]) were made employing the usual assumptions about Poisson-distributed independent pixels.

### 4.2. Experiments with a real detector   

We have devised experiments to estimate the terms expressed in equation (10) for a real p-t-CCD detector, in order to apply the present model for detector response. These experiments were performed on the Rayonix MX300 detector installed at the I24 Microfocus MX beamline at Diamond Light Source (Evans *et al.*, 2007 ▸). For comparison with the simulation, the beamline energy was set to 12 keV.

#### 4.2.1. Determination of the gain   

It is not practical to measure the absorbed photon gain, *G*, directly, but the gain considering all incident photons, *G*′, was determined by comparison of integrated intensities with counts from a scintillation counter. We used a scintillation detector based on a 2.5 mm-thick NaI(Tl) crystal from SCIONIX coupled to an ORTEC photomultiplier base and ORTEC amplifiers. In order to avoid errors due to fluctuations of the source-beam intensity between measurements, the scintillation counter was mounted in front of the MX300 detector and pulse-height spectra were recorded simultaneously with images on the MX300 detector, with the beamline attenuators adjusted to ensure negligible count loss due to dead time of the scintillation counter. All recorded images had full nonuniformity and distortion corrections applied. To ensure a well defined signal on each detector, we exposed a wax sample mounted on the goniometer to produce powder diffraction rings. The wax used was dotriacontane (Aldrich D223107, 97%), as tests showed this to give distinct diffraction rings at low resolution (Brandao-Neto *et al.*, 2010 ▸). The MX300 detector was positioned 1.44 m along the beam path from the sample position, which allowed clear separation of the diffraction rings. Two lead sheets of 2 mm thickness were mounted in front of the detectors to form curtains that were adjusted to produce a vertical slit of approximately 1 mm width, its length forming a secant that cut through the solid angle of a diffraction ring of interest, hence producing two spots at the positions at the top and bottom where the diffraction ring was projected through the slit. The X-rays forming these spots were incident at approximately 2.8° from the detector-face normal. A series of 400 images of 5 s duration was collected first, with the wax sample rotated through the same 80° oscillation range during each exposure, and the scintillation counter removed from position so that both spots were recorded on the MX300 detector. As we did not use a sample spinner, and the width of the slit differed slightly at the positions forming the spots, these images were necessary to determine the relative intensity of the upper and lower spots. The scintillation counter was then moved into position to measure the X-ray photon counts constituting the lower of the pair of spots simultaneously with images of the upper spot. A further 20 exposures were recorded, with exposure times varying between 30 and 120 s. A dark image was determined from the average image of an ensemble of 200 images recorded without beam. As the dark signal was found to be negligible for this detector, subtraction of this averaged dark image was suitable for removing the pedestal offset for images at all exposure lengths used. Following this offset removal, integration was performed by summation of the pixel values in the spot regions of interest. The background count rate for the scintillation counter was determined and used to predict background levels, which were subtracted from the photon counts for each exposure. The photon counts were integrated from the scintillation spectra over the full width of the peak centred at 12 keV. The gain was thus determined by the fit of a linear model between the photon counts and the equivalent intensity of the lower spot, which was inferred from the upper-spot integrated intensity and the relative intensity factor between the upper and lower spots.

#### 4.2.2. Determination of pixel noise   

We wished to partition the total observed variance of a signal into the component due to the cascade-chain response and the component due to pixel noise, consisting of read-out and digitization noise over all of the raw-image pixels contributing to the measurement box of that signal. The measurement box we chose corresponded to the spot formed by the projection of the upper part of the wax diffraction ring through the lead slit. To estimate the pixel-noise component of the variance, we measured the variance of the integrated intensity values within this measurement box on a series of 200 images of 5 s accumulation time but with no exposure to X-rays. We did not record *N*, the number of raw-image pixels corresponding to the measurement box on the corrected image, so we cannot here determine the pixel-noise factor ψ directly. However, knowledge of the total pixel-noise component *N*ψ is sufficient to eliminate this part of the total observed variance of a signal, to leave the part caused by the detector cascade chain.

#### 4.2.3. Variation of response   

In principle, the variation in the detector response could be measured directly over an ensemble of many replicate exposures. It is, however, unfeasible to obtain true replicate exposures, because of instability in the beam intensity. Sequential measurements of intensity form a time series which is nonstationary in mean and variance, owing to gradual drifts in beam intensity as well as abrupt changes caused by, for example, electron-beam injection. During our measurements the synchrotron operated in ‘top-up’ mode, in which the electron-beam current was returned to 150 mA every 600 s. At each 1 s interval throughout the duration of data collection for the set of 400 images described in §[Sec sec4.2.1]4.2.1, we recorded the total current from a QBPM (quad beam position monitor; FMB Oxford Ltd, Oxford, UK) (Alkire *et al.*, 2000 ▸) installed in the beam path. These QBPM readings were background-subtracted in order to find the correct zero level, and the moving average was taken to give a single mean value for each 5 s exposure. A suitable model to describe the time-series trend of the integrated intensity values was found by linear scaling of the averaged QBPM current values to fit the integrated intensity of the spot formed at the intersection of the upper part of the wax diffraction ring and the lead slit. We found it necessary to perform separate linear fits for each gradually changing section of the time series, which were demarcated by abrupt intensity changes such as top-up injections. The aim was empirically to remove, as far as possible, the effects of underlying systematic trends in beam behaviour by breaking the data down into shorter more well behaved sections, where QBPM readings and intensity values could be assumed to be proportional. The data and trend-line fit are described in more detail in the supplementary figure.^1^

The part of the variance of the intensity due to statistical response fluctuations and not due to the intensity trend was estimated by calculating the variance of the residuals between the trend model and the integrated intensity values. Strictly speaking, the variance of the time series is nonstationary, so the variance of the residuals depends in time on the value of the trend. However, the standard deviation of the trend values was only 1.3% of its mean value, so to a reasonable approximation, referring to equations (8) and (10), a signal 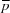 is described by the mean value of the trend, and the variance of that signal 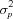 by the variance of the residuals of the measurements from the trend. Of course, this method relies on the accuracy of the trend line. It is likely that the true constant of proportionality between the QBPM values and the integrated intensities also varies to some extent within each separately scaled section, not only between sections as assumed here. In this case it is reasonable to suggest that the estimate of the detector-response variation, and consequently the estimate of the cascade factor γ, are overestimates of their true values.

## 5. Results   

### 5.1. Integration of simulated replicate spot ensembles   

#### 5.1.1. Summation integration   

Sets of replicate spot images were generated and integrated as described in §[Sec sec4.1]4.1. For summation integration, the results are summarized in Table 3 ▸. It can be seen that the variance of the integrated intensity is poorly estimated for each spot under the usual assumptions of independent Poisson pixels. Indeed, as should be expected, for both raw and corrected images the observed sample variance var(*I*_S_) is greater than mean(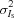) by an amount compatible with the result derived in equation (13). In contrast, the mean estimated error taking into account the cascade model and pixel noise, mean(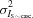), provides a much better agreement with the observed variance. As expected, for corrected images the pixel-noise component of the new model is slightly overestimated because the number of pixels in the corrected-image measurement box is larger than the number of raw-image pixels that contribute to the signal.

It is worth noting that the mean summation integrated intensities for raw images are essentially the same for zones *A* and *B*, but for corrected images they show a small but significant difference. This is because the simulated detector has a uniform response, so performing the nonuniformity correction effectively introduces a small systematic error to the corrected images, rather than removing a larger error due to nonuniform response, as mentioned in §[Sec sec2.4]2.4. For a real detector system, the position-dependent systematic error is exacerbated by fine-grained nonuniformity, discussed in §[Sec sec6.3]6.3.

#### 5.1.2. Profile fitting   

The same sets of images that were integrated by summation integration were also integrated by profile fitting. As can be seen from Table 4 ▸, for spots in both zones the mean estimated error, given by mean(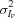), strongly underestimates the observed variance in the integrated intensities. For raw images this is fully due to negligence of the correlation between pixels introduced by the PSF. For corrected images the underestimate is worse, and nonuniformly so, because of the smoothing effect of increased nearest-neighbour pixel correlations which result from the distortion correction. Moving the spot six pixels in *X* and *Y* from zone *A* to zone *B* samples a different correlation structure under the measurement boxes for corrected images. In zone *A*, the observed variance in profile-fitted intensities var(*I*_P_) for corrected images of spots is 3.8 times greater than the mean error estimate mean(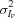). For spots in zone *B* this ratio is 3.4. For raw images, the underestimate of the observed variance is essentially the same in both zones, at 1.7 times. These results demonstrate not only the degree to which the ‘smoothing’ effect introduced by distortion correction further underestimates the true error, but that this underestimate is modulated spatially across the detector face.

The expression for profile-fitting error estimation can be derived by considering the least-squares minimization in matrix form, as presented in Appendix *A* [App appa]. The utility of the general matrix approach is that it is clear how the error estimate for profile fitting can be adapted for the situation where pixels are not independent Poisson sources. A better set of assumptions would populate the off-diagonal elements of the variance–covariance matrix for observations, **M**_*f*_, and ultimately lead to more realistic error estimates for the profile fitted intensity. With a large ensemble of replicate spot images, we had the advantage of being able to calculate **M**_*f*_ based on the real observed variances and covariances across the whole set. For the 1 × 10^5^ spot images in each image ensemble, profile fitting was repeated with a pre-calculated **M**_*f*_, leading to much better profile-fitting error estimates (see Table 4 ▸). In this case, **M**_*f*_ consisted of the observed variances and covariances for pixels within the measurement box, with covariance values calculated up to seven neighbouring pixels deep in *X* and *Y*. This recovers essentially all of the variance due to covariance elements for the raw images, and the majority of the co­variance for the corrected images.

### 5.2. Comparison with a real detector   

In order to study the response of a real p-t-CCD detector in terms of the new model presented here, we performed the experiments described in §[Sec sec4.2]4.2. In particular, we wished to determine the cascade factor γ, which encapsulates the excess variance caused by the detection cascade compared with a Poisson distribution. In order to determine γ, we needed first to quantify the other parameters expressed in equation (10) and then use this to model the observed variation of a signal measured on the detector.

The gain for all incident photons, *G*′, for this detector was determined in two steps, first by determining the relative intensity of two spots integrated in regions of images from the area detector, and second by simultaneously recording images of one of those spots whilst recording the intensity of the other with a scintillation counter. The estimated error on the gain measurement was therefore combined from the quality of the linear fits from both stages. Using this method we measured the gain to be *G*′ = 1.22 (5) ADU per incident 12 keV X-ray photon. By assuming a phosphor quantum absorption of 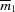 = 0.85, which is the same as used in the simulation, our estimate for the gain considering only absorbed X-rays was *G* = 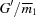 = 1.44 ADU per interacting 12 keV X-ray photon.

The total expected pixel noise for the region of interest was measured to be *N*ψ = 5454 ADU^2^. This region consisted of 1144 pixels on the corrected image, although the number of raw image pixels, *N*, over which the signal was distributed was not determined.

To measure the response variation of the detector, the signal we investigated was the sum of the pixel values, after offset correction, in a region formed by the projection of a diffraction ring through a lead slit. Although this feature is unlike a typical Bragg spot, it may be used to investigate the variance response using the general formula given by equation (10). In this case the magnitude of the measured signal was 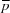 = 11 926 ADU with an estimated variance of 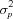 = 29 608 ADU^2^. Substituting these values and the experimentally determined estimates for *G* and *N*ψ into equation (10) allows an estimate of the cascade factor for this detector as ψ = 1.41. As described in §[Sec sec4.2.3]4.2.3, inasmuch as the trend-line fit to the integrated intensity data deviates from the true trend, this value may overestimate the true value of γ.

## 6. Discussion   

### 6.1. The effect of non-Poisson response on error estimates   

Typical error estimates of the summation integrated intensity assume that each pixel independently obeys Poisson statistics. The use of a cascade model for a p-t-CCD detector has allowed the construction of a summation integration intensity error estimate formulation that properly takes into account the combined statistical response of all elements in the detection chain, including a contribution from the number of pixels the integration is performed over, due to read-out noise and digitization error. The simulation results presented in §[Sec sec5.1.1]5.1.1 show that this more comprehensive model provides a better estimate of the observed random error in summation integrated spot intensities.

Our experimental data demonstrated the degree to which a Poisson model underestimates the variance of a signal recorded on a real p-t-CCD detector. Measurements of the parameters of a cascade model were made for the real CCD detector, showing how this model could be applied in future for error estimates in integration. However, experimental determination of the absolute value of the cascade factor γ and the absorbed photon gain *G* in the way described requires knowledge of the phosphor quantum absorption 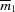, which here was simply assumed to be equal to the simulated value. In addition, the parameters γ, *G* and 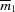 are all functions of the X-ray energy and angle of incidence, which should be taken into consideration for a full characterization of a real detector.

Interestingly, comparison of the cascade factor calculated for the simulation (γ = 1.54) with that determined experimentally for the Rayonix MX300 detector installed at the I24 beamline (γ = 1.41) suggests that the simulated detector has pessimistic noise properties compared with this real detector. Differences between the simulated detector and real p-t-CCD detectors are expected, as even though our simulation was based on the MX series of detectors from Rayonix, the parameters used within the simulator to describe elements of the cascade chain were not experimentally determined but derived from reasonable estimates based on known properties of the components. In particular, the simulated phosphor screen differs from those used in real commercially available p-t-CDD detectors in that no reflective coating was modelled. A reflective layer on the outer side of the phosphor coat increases the signal-to-noise ratio of the phosphor screen by reflecting backscattered light towards the FOT face (Nishikawa *et al.*, 1989 ▸). This is a possible explanation for the difference between our model and experimental observations.

### 6.2. The effect of pixel correlations on error estimates   

Summation integration error estimates avoid the need to consider pixel correlations, because summation over all the pixels in the peak region can potentially recover the entire original signal incident on the detector, irrespective of how that signal was apportioned between those pixels. Nevertheless, any integration procedure in which the profile of the spot is important clearly necessitates a treatment of pixel correlations. In profile fitting, integration is performed by a least-squares fit of a standard or reference spot profile to the observed data. The profiles of partially recorded reflections may differ significantly from the standard profile. For this reason, fully and partially recorded reflections are treated differently by *MOSFLM*, and error estimates in the case of partially recorded reflections are taken from the summation integration error estimation formula (Leslie, 1999 ▸). For profile fitting of fully recorded reflections, error estimates are based on the quality of fit of the standard profile. Correlations between pixels effectively smooth an image by reducing the pixel-to-pixel variance. The standard approach to profile fitting is in fact a method of fitting a smoothed model (the profile) to smoothed data. If the degree of correlation between pixels is not addressed, the fit can appear artificially good. This has been demonstrated by our simulations (see §[Sec sec5.1.2]5.1.2).

Although we have shown the importance of considering realistic covariances between pixels for profile-fitting integration, this is difficult to put into practice because of the need to obtain estimates of the covariances between pixels from a single image. It is our intention further to investigate methods for formulating accurate covariance estimators for images from CCD detectors. It appears likely that an effective method will require information about the local smoothing caused by the distortion correction, and the point-spread function of the phosphor and other optical elements, plus an estimate of the profile of the spot at the detector face. It may be possible to obtain suitable estimates of the latter by deconvolution of the measured profile, or by *ab initio* prediction (Schreurs *et al.*, 2010 ▸).

### 6.3. Position-dependent systematic errors   

In this work, we have concentrated on improving the accuracy of estimates of the random errors associated with data from p-t-CCD detectors. Despite this, it is worth discussing the various sources of instrumental systematic errors that result from the detection or correction procedures, as these errors are often significant.

For real detectors, the ADU content of dark images results from accumulated dark current, plus ADC bias, read-out noise and spurious zingers. If a dark image is to be used for correction of all the raw images in a data set, then the random noise and anomalous outliers should be suppressed to reduce the systematic error at each pixel that dark subtraction introduces. Zingers are usually removed using an algorithm that identifies outliers by comparison with a second image (Barna *et al.*, 1999 ▸), but the dezingered dark image is still subject to read-out noise. Although the magnitude of the read-out noise is usually small, for weak reflections recorded over a large number of pixels the total error introduced in the intensity by dark subtraction could be significant. For this reason, it is good practice to collect a large number of dark images of the required exposure length and average them to reduce the read-out noise contribution.

Real p-t-CCD detectors exhibit a nonuniform response due to phosphor-screen variations, obliquity of incidence (as this changes the apparent phosphor thickness), FOT inhomogeneities and variations in the CCD sensor response (Barna *et al.*, 1999 ▸). The effect of this nonuniform response is to produce a systematic error in the recorded intensities. The removal of this systematic error is limited by the accuracy to which the normalizing flat-field image has been determined, such that if the flat field is known to within 1% error, pixel-value accuracy in the corrected image can only be given to the same level or worse. As with the systematic error introduced by dark subtraction, this should be considered when evaluating detector performance.

It may seem at first that the magnitude of this systematic error can be reduced towards zero by improving the statistical quality of the flat-field image. However, there is a more pernicious problem when a flat-field correction is performed on diffraction data. Inhomogeneities of response of the phosphor on a scale smaller than that of the point spread will be averaged out in the flat-field by the smoothing effect of the point-spread function. In contrast, sharp sources, such as diffraction spots, sample only the local phosphor response, leaving the effect of comparatively ‘hot’ or ‘cold’ regions visible in the resulting images. Correction factors derived from a flat field are therefore of limited accuracy (Tate *et al.*, 1995 ▸). Similarly, subpixel scale granularity of the combined phosphor and taper response becomes important when the source signal has features with significant contrast on the scale of a pixel. Clearly, a point source incident at different positions within the same pixel region at the detector face will result in different pixel values if the variation in subpixel response of the optical chain is significant (Gruner *et al.*, 2002 ▸). The net effect of both the point-spread smoothing and pixel discretization is a position-dependent nonuniformity, which is worse the sharper the source feature is. This manifests as a systematic error that increases the scatter of measured intensities of crystallographic symmetry-related reflections, and even between the φ slices of partially recorded reflections, if the profile varies enough between the slices.

It is not possible to generalize the severity of this effect for diffraction data, as it is strongly dependent on the spot size and spot-profile gradient. Nevertheless, we have observed errors greater than 1% of the intensity in tests on real p-t-CCD detectors for data representative of typical diffraction spots. For strong spots where the random errors suggest a good relative precision, the size of this systematic error therefore dominates the total error. The presence of this effect may explain why the instrumental error factor from *MOSFLM* is useful for data from p-t-CCD detectors, despite its physical justification being based on densitometry of X-ray films. In order to understand better the size and character of this position-dependent systematic error, we intend to investigate it in detail by experiment. Even when a justifiable formulation for the contribution of this error is produced, we maintain that it is preferable to keep this separate from the random measurement error, as a step towards a more sophisticated scheme of error awareness and tracking.

## 7. Conclusions   

Macromolecular crystallography is a technique in which it is often paramount to extract small signals from noisy data in order to solve a particular scientific problem. The assignment of realistic errors to recorded intensities has an impact on all stages of structure determination and refinement. Clearly, the initial measurement errors propagate through data-processing steps and determine the limit on the accuracy of derived quantities. Nevertheless, the model for measurement error at the point of integration is commonly inadequate and realistic errors are only determined at the scaling step, in which all components of the experimental error are combined in a composite error model. This makes it difficult to break down the total error into its components. It is hoped that a more detailed model of the diffraction experiment will allow the proper assignment of uncertainties at all points. Once all known error sources are accounted for, it will be clear which areas contribute the most to the degradation of signal to noise and whether any part of the diffraction physics remains unaccounted for.

In this work, we have looked specifically at the measurement errors associated with the integration of images obtained from p-t-CCD detectors, currently the most popular type of diffraction image detector used at macromolecular crystallography beamlines. Using a simulation of a p-t-CCD detector and integration routines, we have shown how the assumptions of Poisson statistics and pixel independence are unfounded and lead to underestimates of the true random error in measurements. These underestimates have previously been enlarged using a heuristic instrumental error factor, but this is intended to model an effect different from the response of a p-t-CCD detector. This effect is not linearly proportional to intensity and has a much greater impact for strong reflections. The inflated error estimates may mitigate against underestimates in the case of strong reflections, but fail to capture the distinction between random errors from the source and the detector response, and systematic errors that could in principle be identified and corrected. We have shown how summation integration procedures can be readily modified to take into account properly the detector response and noise at each pixel. However, profile fitting on distortion-corrected images incurs a spatial dependence caused by a nonuniform pattern of correlation between pixels that cannot be corrected by a global scale factor. Methods to produce accurate profile-fitting error estimates based on local properties of the image are the subject of ongoing investigation.

Ultimately, it is hoped that integration procedures will correctly assign measurement errors consisting of the statistical noise of the input signal combined with the appropriate local detector-response noise, opening the way to quantification of other noise sources which become apparent when data are put on a common scale. These noise sources should be described fully by a detailed model of the physics of the diffraction experiment, in which effects such as sample absorption, X-ray background structure and detection physics are accounted for. By explaining as much of the experimental data as possible by a justifiable model we hope to ensure the best treatment of this data, improving results particularly for marginal cases of structure solution.

## Supplementary Material

. DOI: 10.1107/S0021889810033418/ea5116sup1.pdf
Supplementary figure

## Figures and Tables

**Table 1:** Phosphor simulation details

Phosphor material	Gd_2_O_2_S:Tb
Density	7.34 g cm^−3^
Packing density	50%
Coating weight	14 mg cm^−2^
Grain size	5 µm
Intrinsic efficiency	0.15
Incident X-ray energy (monoenergetic)	12 keV
Generated light wavelength (monoenergetic)	545 nm
Phosphor point spread† FWHM	<40 µm
Phosphor point spread FW10%M	<100 µm

† Phosphor point spread was determined as described for Fig. 1 ▸.

**Table 2:** Parameters of the simulation

Item		Source	Value
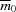	Number of incident X-ray photons	Poisson-distributed signal	
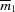	Fraction of incident X-ray photons that interact	Binomial success probability, equal to the phosphor quantum absorption	0.85
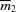	Mean quantum amplification of phosphor	Simulated scintillation spectrum	281.5 photons
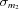	Standard deviation of the quantum amplification ofthe phosphor	Standard deviation of the simulated scintillation spectrum	163.3 photons
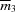	Overall transmission	Binomial success probability, product of: 50% FOT acceptance, 14% FOT transmittance, 70% FOT to CCD fibre-optic stub acceptance, 35% quantum efficiency of CCD	0.01715
*N*	Number of pixels signal is recorded over	Determined by integration procedure	
σ_r_	CCD read-out noise	Normal distribution	10 electrons r.m.s.
*g*_ADC_	Number of electrons per ADU	Fixed constant	0.2

**Table 3:** Summation integration of 1 × 10^5^ simulated spots In the column of equation references, *M* indicates a reference to *MOSFLM*, described by Leslie (1999 ▸). The stated precisions are given by the standard errors of the estimators.

			Zone *A*		Zone *B*	
Statistics over 1 × 10^5^ samples		Refer to equation	Raw	Corrected	Raw	Corrected
Summation integration intensity distribution	mean(*I*_S_) (ADU)	Equation (15)	8205.3 (4)	8254.0 (4)	8204.7 (4)	8206.4 (4)
	var(*I*_S_) (ADU^2^)	Equation (15)	18240 (80)	18890 (80)	18060 (80)	18640 (80)
Variance estimates	mean( 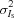 ) (ADU^2^)	Equation (11) in *M*	11651.0 (4)	12385.3 (4)	11640.1 (4)	12367.6 (4)
	mean( 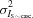 ) (ADU^2^)	Equation (18)	18761.8 (6)	20302.2 (6)	18745.0 (6)	20274.8 (6)

**Table 4:** Profile fitting of 1 × 10^5^ simulated spots The stated precisions are given by the standard errors of the estimators.

			Zone A		Zone B	
Statistics over 1 × 10^5^ samples		Refer to equation	Raw	Corrected	Raw	Corrected
Profile-fitted intensity distribution	mean(*I*_P_) (ADU)	Equation (15)	8181.8 (4)	8240.3 (4)	8217.7 (4)	8209.8 (4)
	var(*I*_P_) (ADU^2^)	Equation (15)	13640 (60)	13770 (60)	13520 (60)	13420 (60)
Variance estimates	mean( 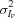 ) (ADU^2^)	Equation (39)	8137 (2)	3598 (1)	8170 (2)	3898 (1)
	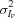 with **M**_*f*_ † (ADU^2^)	Equation (18)	13669	13305	13530	12954

† In this case, the variance–covariance matrix of observations, **M**_*f*_, was pre-calculated from the set of 1 × 10^5^ samples, rather than estimated individually for each.

## Footnotes

1 Supplementary material discussed in this paper is available from the IUCr electronic archives (Reference: EA5116). Services for accessing these data are described at the back of the journal.

## Appendix A Error estimates for profile-fitted intensities

Here a derivation of the error estimate associated with a profile-fitted intensity is given, closely following the terminology presented by Giacovazzo (2002 ▸). Assuming an appropriate standard profile *P* is available, the spot intensity is evaluated by determining a scale factor *K* from the fit of this profile to the data, then summing over all pixels *i* in the profile:

*K* is determined at the same time as the background plane parameters *a*, *b* and *c*, with the background plane defined in the same way as for summation integration. A value for  is obtained from the least-squares treatment as detailed in the following text.

The task of fitting the profile and background parameters to the data can be formulated by considering the least-squares minimization of the residual

where *w*_*i*_ =  is the weight at pixel *i*. This can be expressed for the general case in matrix form by defining

with observations

parameters

the design matrix

and the variance–covariance matrix

With these definitions, the residual *S* can be written

By further defining

the normal equations, which come from the partial derivatives of the residual *S* with respect to the parameters, can be expressed by

such that the least-squares solution for  (the best estimate of **X**) is

The variance  is contained in the relevant element of **M**_*x*_, the variance–covariance matrix for parameters. It can be shown (Giacovazzo, 2002 ▸) that

Thus, the errors in the parameters of the model can be related to the errors in the observations, **M**_*f*_. Specifically for the profile-fitting error estimate of equation (20),

The accuracy of the error estimate in the profile-fitted intensity falls to the correct estimation of the covariance values σ_*i*,*j*_. In *MOSFLM*, it is assumed that the pixels are independently distributed according to a Poisson distribution. The off-diagonal (covariance) elements of **M**_*f*_ are set to zero, while the variance elements are calculated within a scale factor *K*_v_ as

such that

The scale factor *J* for profile values is estimated by comparison with the summation integration intensity using

*K*_v_, the scale of the variance matrix **M**_*f*_, is unknown, but can be estimated from the goodness of fit of the least-squares procedure using the reduced chi-squared statistic

where *n* gives the number of data points and *m* the number of parameters. In this case,

Thus, the full expression for the estimated error in the profile-fitted intensity is given by

therefore

which coincides with the expression given in *MOSFLM* (Leslie, 1999 ▸).
