# Supplementary material for: Estimation of errors in diffraction data measured by CCD area detectors
Source: J Appl Crystallogr. 2010 Oct 1;43(Pt 6):1356–71. doi: 10.1107/S0021889810033418 (PMC4786054; doi:10.1107/S0021889810033418)
Supplement: Supplementary file 1 [file j-43-01356-sup1.pdf]

Supplementary material

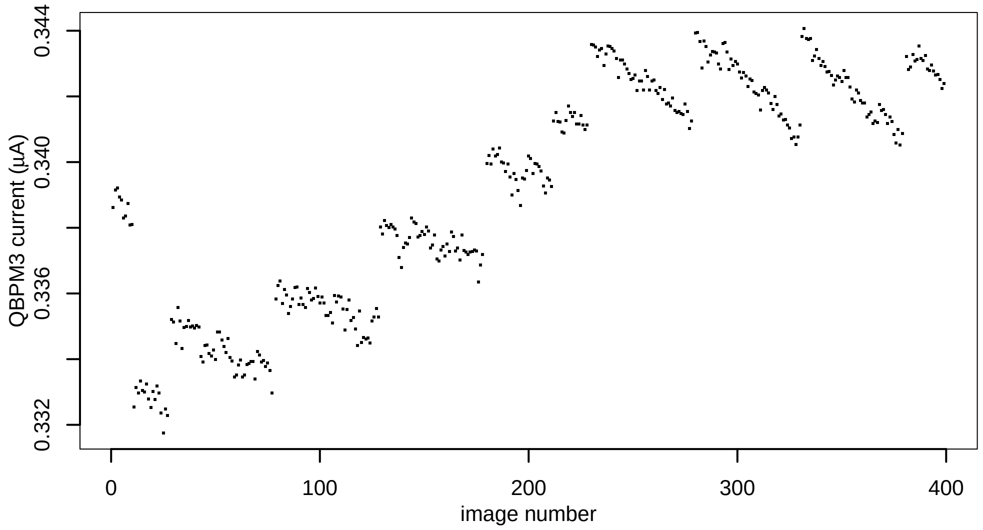

(a)

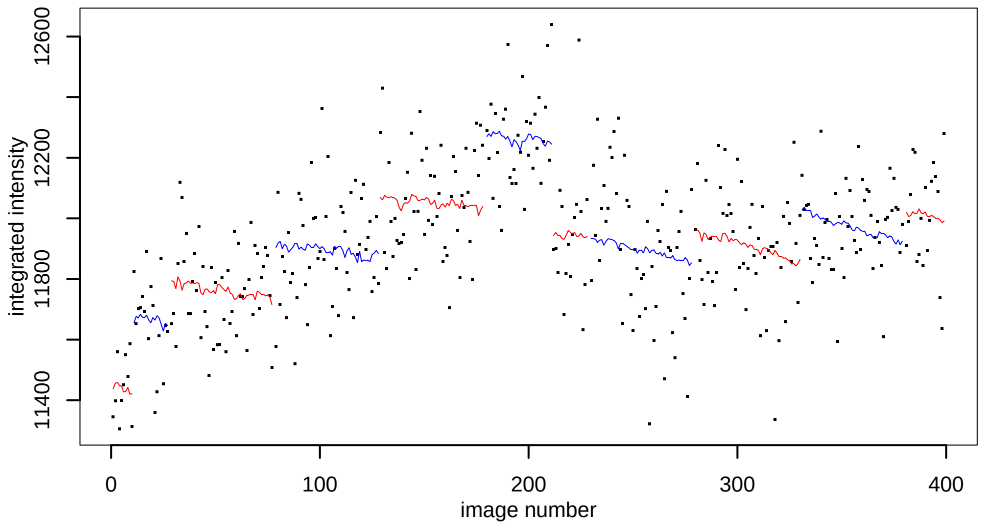

(b)

Figure S1

Here the data used to estimate the response variation, and consequently the cascade factor γ, for a real p-t-CCD detector is shown and the method used to fit a trend line to it is explained. *(a)* The total current recorded by a QBPM (quad beam position monitor) has been averaged to give a single value for each exposure and is plotted against corresponding image number. Outliers affected by readings taken during abrupt intensity changes have been removed. Most of these abrupt changes are caused by electron beam injection at regular 10 minute intervals, however additional discontinuities are present after image 10 and after image 211, the cause of which are unknown. *(b)* The summation integrated intensity of a spot formed as described in the main text is plotted against image number. We wished to use the information recorded by the QBPM in order to form a trend line that would account for the gradual drifts in integrated intensity, whilst also containing information about fluctuations on the image-to-image time scale. As no global scale factor could be found to adequately relate the data shown in *(a)* to the integrated intensities, individual scale factors for each section between abrupt changes have been determined in order to form a piecewise trend line for the data, which is plotted with sections alternately coloured in red or blue. The presence of abrupt changes of QBPM current that are not reflected by proportionate changes in the integrated intensity warrants attention. The likely cause is movement of the X-ray beam relative to optical components positioned between the QBPM and the area detector. These components include a set of vertical and horizontal microfocusing mirrors, the on-axis viewing system and a collimating aperture. The exact cause of the beam movement is as yet unknown but could be either upstream optical components such as the monochromator or the X-ray source itself. At the time of performing this experiment an accurate beam intensity diagnostic closer to the sample position was not available.
